# Clinical and Genetic Profile of X-Linked Agammaglobulinemia: A Multicenter Experience From India

**DOI:** 10.3389/fimmu.2020.612323

**Published:** 2021-01-15

**Authors:** Amit Rawat, Ankur Kumar Jindal, Deepti Suri, Pandiarajan Vignesh, Anju Gupta, Biman Saikia, Ranjana W. Minz, Aaqib Zaffar Banday, Rahul Tyagi, Kanika Arora, Vibhu Joshi, Sanjib Mondal, Jitendra Kumar Shandilya, Madhubala Sharma, Mukesh Desai, Prasad Taur, Ambreen Pandrowala, Vijaya Gowri, Sneha Sawant-Desai, Maya Gupta, Aparna Dhondi Dalvi, Manisha Madkaikar, Amita Aggarwal, Revathi Raj, Ramya Uppuluri, Sagar Bhattad, Ananthvikas Jayaram, Harsha Prasad Lashkari, Liza Rajasekhar, Deenadayalan Munirathnam, Manas Kalra, Anuj Shukla, Ruchi Saka, Rajni Sharma, Ravinder Garg, Kohsuke Imai, Shigeaki Nonoyama, Osamu Ohara, Pamela P. Lee, Koon Wing Chan, Yu-Lung Lau, Surjit Singh

**Affiliations:** ^1^ Allergy Immunology Unit, Department of Pediatrics, Advanced Pediatrics Centre, Postgraduate Institute of Medical Education and Research, Chandigarh, India; ^2^ Department of Immunopathology, Postgraduate Institute of Medical Education and Research, Chandigarh, India; ^3^ Department of Immunology, B. J. Wadia Hospital, Mumbai, India; ^4^ Bone Marrow Transplant Unit, B. J. Wadia Hospital, Mumbai, India; ^5^ Department of Pediatric Immunology and Leukocyte Biology, ICMR-National Institute of Immunohematology, K.E.M Hospital, Mumbai, India; ^6^ Department of Clinical Immunology and Rheumatology, Sanjay Gandhi Postgraduate Institute of Medical Sciences, Lucknow, India; ^7^ Department of Pediatric Hematology, Oncology, Blood and Marrow Transplantation, Apollo Hospitals, Chennai, India; ^8^ Division of Pediatric Immunology and Rheumatology, Department of Pediatrics, Aster CMI Hospital, Bengaluru, India; ^9^ Neuberg Anand Reference Laboratory, Bengaluru, India; ^10^ Department of Paediatrics, Kasturba Medical College, Mangalore, Manipal Academy of Higher Education, Manipal, India; ^11^ Department of Clinical Immunology and Rheumatology, Nizam's Institute of Medical Sciences, Hyderabad, India; ^12^ Department of Pediatric Hematology Oncology and Bone Marrow Transplant, Kanchi Kamakoti Childs Trust Hospital, Chennai, India; ^13^ Department of Pediatric Hematology, Oncology and BMT, Sir Ganga Ram Hospital, New Delhi, India; ^14^ Niruj Rheumatology Clinic, Ahmedabad, India; ^15^ Department of Community Pediatrics, Perinatal and Maternal Medicine, Graduate School of Medical and Dental Sciences, Tokyo Medical and Dental University (TMDU), Tokyo, Japan; ^16^ Department of Pediatrics, National Defense Medical College, Tokorozawa, Japan; ^17^ Department of Applied Genomics, Kazusa DNA Research Institute, Kisarazu, Japan; ^18^ Department of Paediatrics and Adolescent Medicine, Queen Mary Hospital, University of Hong Kong, Hong Kong, China

**Keywords:** arthritis, *BTK* gene, intravenous immunoglobulin, neutropenia, X-linked agammaglobulinemia

## Abstract

**Background:**

There is paucity of literature on XLA from developing countries. Herein we report the clinical and molecular profile and outcome in a multicenter cohort of patients with XLA from India.

**Methods:**

Data on XLA from all regional centers supported by the Foundation for Primary Immunodeficiency Diseases (FPID), USA and other institutions providing care to patients with PIDs were collated. Diagnosis of XLA was based on European Society for Immunodeficiencies (ESID) criteria.

**Results:**

We received clinical details of 195 patients with a provisional diagnosis of XLA from 12 centers. At final analysis, 145 patients were included (137 ‘definite XLA’ and eight ‘probable/possible XLA’). Median age at onset of symptoms was 12.0 (6.0, 36.0) months and median age at diagnosis was 60.0 (31.5, 108) months. Pneumonia was the commonest clinical manifestation (82.6%) followed by otitis media (50%) and diarrhea (42%). Arthritis was seen in 26% patients while 23% patients developed meningitis. Bronchiectasis was seen in 10% and encephalitis (likely viral) in 4.8% patients. *Pseudomonas aeruginosa* was the commonest bacterial pathogen identified followed by *Streptococcus pneumoniae*, *Staphylococcus aureus* and *Klebsiella pneumoniae*. Molecular analysis revealed 86 variants in 105 unrelated cases. Missense variants in *BTK* gene were the most common (36%) followed by frameshift (22%) and nonsense variants (21%). Most pathogenic gene variants (53%) were clustered in the distal part of gene encompassing exons 14–19 encoding for the tyrosine kinase domain. Follow-up details were available for 108 patients. Of these, 12% had died till the time of this analysis. The 5-year and 10-year survival was 89.9% and 86.9% respectively. Median duration of follow-up was 61 months and total duration of follow-up was 6083.2 patient-months. All patients received intravenous immunoglobulin (IVIg) replacement therapy. However, in many patients IVIg could not be given at recommended doses or intervals due to difficulties in accessing this therapy because of financial reasons and lack of universal health insurance in India. Hematopoietic stem cell transplant was carried out in four (2.8%) patients.

**Conclusion:**

There was a significant delay in the diagnosis and facilities for molecular diagnosis were not available at many centers. Optimal immunoglobulin replacement is still a challenge

## Introduction

X-linked agammaglobulinemia (XLA) is one of the most frequent inborn errors of immunity (IEI). Patients typically present with recurrent sinopulmonary and gastrointestinal infections (1). Meningitis, sepsis, arthritis, skin and soft tissue infections and enteroviral encephalitis are the other common clinical manifestations seen in these patients. Autoimmune and inflammatory complications have also been reported rarely (2). XLA results from loss of function variants in Bruton Tyrosine Kinase (*BTK)* gene (3, 4) which is located on long arm of X chromosome (Xq21.3 to Xq22) (5). Pathogenic variants in *BTK* gene lead to maturation arrest of developing B-lymphocytes in the bone marrow at pre-B cell stage (3, 4). Diagnosis is based on presence of pan-hypogammaglobulinemia and absence of mature B-lymphocytes in peripheral blood. Confirmation of diagnosis requires evidence of reduced expression of Btk protein on flow cytometry (or on Western blot) and *BTK* gene sequencing.

There is paucity of literature on XLA from developing countries. This is largely because of lack of awareness and dearth of appropriate diagnostic facilities (6–8). We reported a single center experience on 36 patients with XLA from Chandigarh, India in 2016 (9). Apart from this, literature on XLA from India is limited to single cases report and a small case series (10–19). Clinical profile of patients with XLA may vary from one country to another (20). This could be because of regional differences in infection profile or differences in genetic profile among populations. For instance, vaccine associated paralytic poliomyelitis (VAPP) has been reported from countries where live oral poliovirus is still being used (21). Survival rates of patients with XLA beyond 20 years vary from 70% in developed regions of the world (e.g. North America, Europe and Australia) to 40% in developing countries in Asia and Africa (20, 22).

Herein we report the clinical and molecular profile and outcome in a multicenter cohort of patients with XLA from India. This is the first attempt at nationwide data collection on XLA.

## Methods

**Figure 1:**
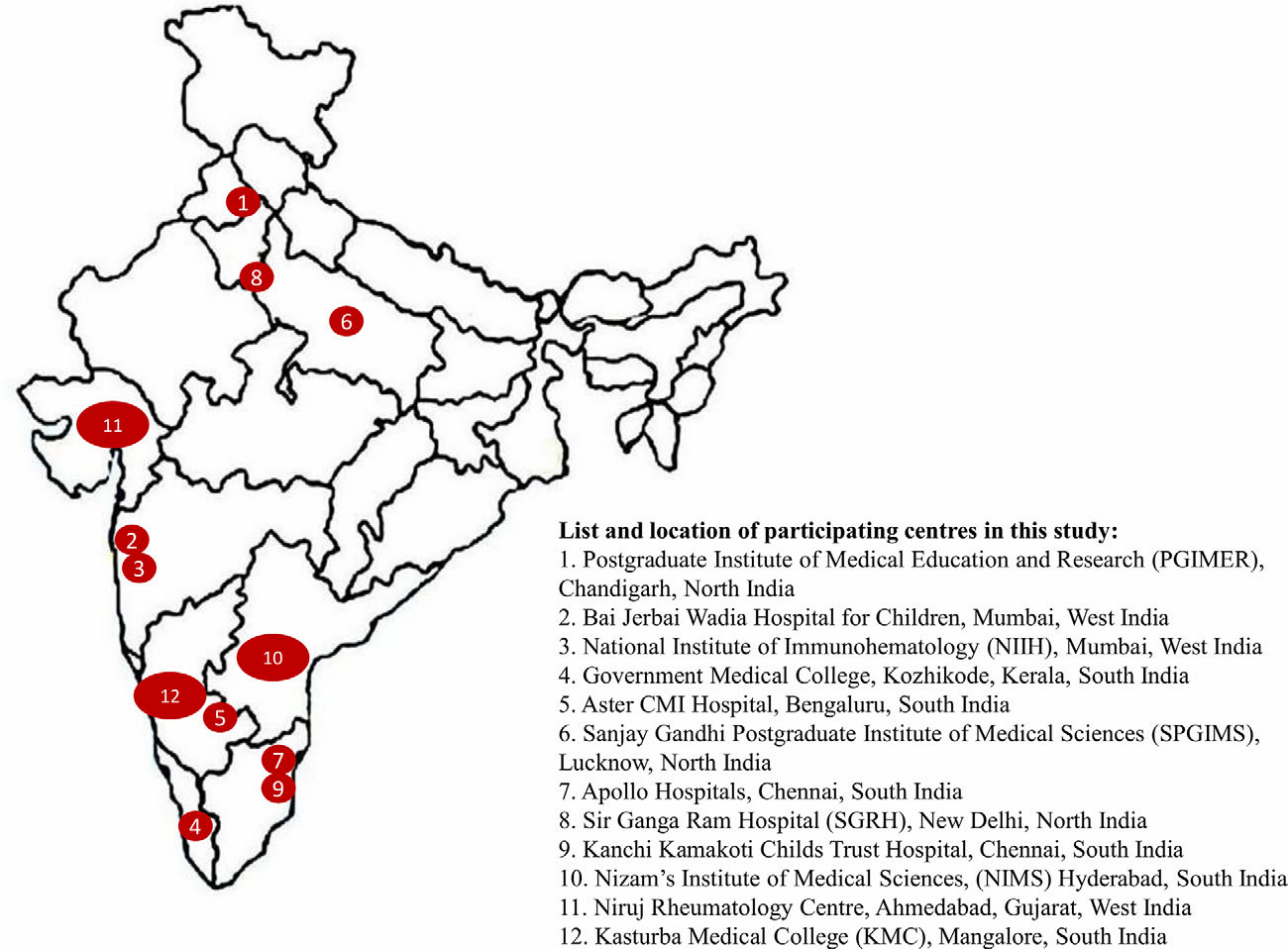
Shows list and locations of centers that participated in this multicenter study.

Data on XLA from all regional centers supported by the Foundation for Primary Immunodeficiency Diseases (FPID), USA and other institutions providing care to patients with PIDs were collated. These centers included Postgraduate Institute of Medical Education and Research (PGIMER), Chandigarh, North India; Bai Jerbai Wadia Hospital for Children, Mumbai, West India; National Institute of Immunohematology (NIIH), Mumbai, West India; Government Medical College, Kozhikode, Kerala, South India; Aster CMI Hospital, Bengaluru, South India; Sanjay Gandhi Postgraduate Institute of Medical Sciences (SPGIMS), Lucknow, North India; Apollo Hospitals, Chennai, South India; Kasturba Medical College (KMC), Mangalore, South India; Sir Ganga Ram Hospital (SGRH), New Delhi, North India; Kanchi Kamakoti Childs Trust Hospital, Chennai, South India; Nizam’s Institute of Medical Sciences, (NIMS) Hyderabad, South India, and Niruj Rheumatology Clinic, Ahmedabad, Gujarat, West India (**Figure 1**). All centers were contacted *via* email and requested to provide details of their patients with XLA on a pre-designed Microsoft Excel sheet. Details included demographic information, clinical manifestations, immunological investigations, genetic diagnosis, treatment and follow-up. The central data collection center was PGIMER, Chandigarh (Review board no.: 194-20)

Assay of serum immunoglobulins (IgG, IgA, and IgM) are now being carried out by nephelometry or turbidimetry at most centers in India. In the past, radial immunodiffusion (RID) was the preferred method for assay. Lower limit for detection of serum immunoglobulins varied depending on type of technique and type of kit used. For instance, in Chandigarh the lower limits for detection of IgG, IgA, and IgM are 2.02 gm/l, 0.17 gm/l, and 0.25 gm/l. Serum immunoglobulins were reported to be ‘below lower limit of detection’ in several patients and absolute value was not given. Serum IgE was assessed using enzyme linked immunosorbent assay (ELISA). Lymphocyte subset analysis and Btk protein estimation on peripheral blood monocytes was performed using flow cytometry (9).

*BTK* gene analysis was carried out using Sanger sequencing, targeted next generation sequencing or whole exome sequencing depending on availability and preference at individual centers.

Diagnosis of XLA was based on European Society for Immunodeficiencies (ESID) criteria (23). The ESID criteria defines ‘*Definitive XLA’* as a male patient with less than 2% CD19+ B cells and at least one of the following: 1) Mutation in *BTK* gene; 2) Absent Btk mRNA on northern blot analysis of neutrophils or monocytes; 3) Absent Btk protein in monocytes or platelets; 4) Maternal cousins, uncles or nephews with less than 2% CD19+ B cells. ‘*Probable XLA’* is defined as a male patient with less than 2% CD19+ B cells in whom all of the following are positive: 1) Onset of recurrent bacterial infections in the first 5 years of life; 2) Serum IgG, IgM and IgA more than 2SD below normal for age; 3) Absent isohemagglutinins and /or poor response to vaccines; 4) Other causes of hypogammaglobulinemia have been excluded. ‘*Possible XLA’* is defined as a male patient with less than 2% CD19+ B cells in whom other causes of hypogammaglobulinemia have been excluded and at least one of the following is positive: 1) Onset of recurrent bacterial infections in the first 5 years of life; 2) Serum IgG, IgM and IgA more than 2 SD below normal for age; 3) Absent isohemagglutinins.

For purposes of the present study, male patients with hypogammaglobulinemia and decreased or absent B cells and X-linked family history (i.e. involvement of male siblings, maternal cousins, maternal uncles or nephews) were labeled as *‘probable’* or *‘possible’* XLA. Patients with hypogammaglobulinemia and low B cell numbers who had no suggestive X-linked family history and in whom Btk protein expression was either not performed or was normal and *BTK* gene sequencing could not be carried out, were excluded from final analysis.

### Statistical Analysis

Data were obtained on a predesigned worksheet (Excel, Microsoft Office) which were analyzed using the Statistical Package for the Social Sciences software (SPSS, version 23, IBM Corporation). As most variables had a non-parametric distribution, continuous variables were summarized as median (25^th^, 75^th^ percentile) [n], where ‘n’ represents the total number of patients for whom the said data were available. Nominal variables were expressed as percentages [x] [n=y], where ‘x’ represents the absolute number and ‘n’ represents the total number of patients for whom the said data were available.

## Results

**Figure 2:**
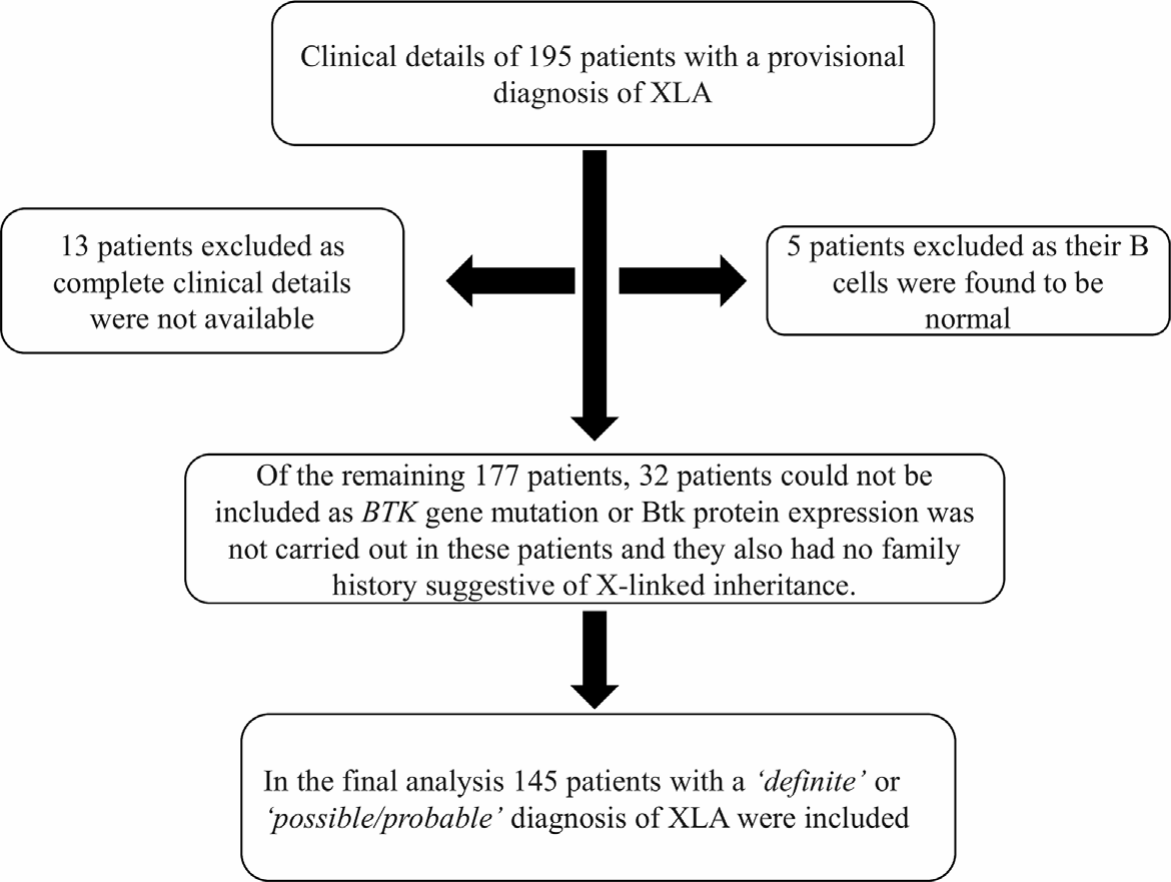
Schematic diagram showing inclusion and exclusions of cases in the study. Abbreviations: XLA, X-linked agammaglobulinemia; BTK, Bruton tyrosine kinase.

In this multicenter cohort from 12 centers in India, we initially received clinical details of 195 patients who had hypogammaglobulinemia and a provisional diagnosis of XLA was considered by the treating physician. On initial analysis, five patients were excluded as their B cell numbers were found to be normal; 13 patients had to be excluded as complete clinical details were not available. Of the remaining 177 patients, 32 patients could not be taken up for final analysis as *BTK* gene mutation or Btk protein expression had not been carried out in these patients. They also did not have family history suggestive of X-linked inheritance. Therefore, in the final analysis, 145 patients were included (137 *‘definite XLA’* and eight *‘probable/possible XLA’*) (**Figure 2**).

### Clinical Profile

Median age at onset of symptoms was 12.0 (6.0, 36.0) months [n=130]. Median age at diagnosis was 60.0 (31.5, 108) [n=144] months. Median delay in diagnosis was 42.0 (15.8, 72.0) [n=130] months. 59.2% [77] [n=130] patients had onset of symptoms on or before the age of 1 year; however, only 6.2% [8] [n=130] were diagnosed before their first birthday. Details about family history were available in 87 patients. Of these 52 (60%) had a family history suggestive of an X-linked inheritance. There was no significant difference in the duration of diagnostic delay or the outcomes when patients with family history were compared with those who lacked a family history. In children diagnosed in the 1^st^ year of life, family history was negative in 50% [4] [n=8], positive in 25% [2] [n=8], and unavailable in the rest [2].

**Figure 3:**
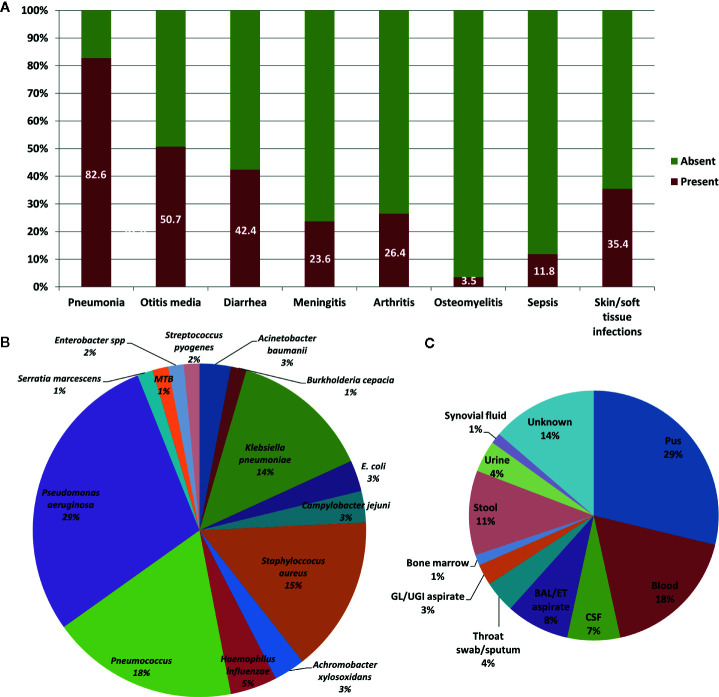
Clinical profile of patients with X-linked agammaglobulinemia (XLA). **(A)** Bar chart showing infections that were seen in patients with XLA. **(B)** Pie chart showing various pathogenic organisms that were isolated from patients with XLA. **(C)** Pie chart showing various techniques used for isolating the pathogenic organisms.

Pneumonia was the commonest clinical manifestation seen in 82.6% [119] [n=144] patients. Complications of pneumonia in form of empyema was seen in 13.4% [16] [n=119] patients. Other common manifestations were recurrent otitis media (50.7% [73] [n=144]), recurrent diarrhea (42.4% [61] [n=144]), and skin and soft tissue infections (35.4% [51] [n=144]) including suppurative lymphadenitis. Arthritis was seen in 26% (36 [n=144]) patients while 23% (34 [n=144]) patients developed meningitis. Bronchiectasis was seen in 10% (14 [n=144]) patients. Encephalitis (likely viral) was observed in 4.8% (7 [n=144]) patients. Clinical profile of our cohort is summarized in **Figure 3A**.

Neutropenia (defined as absolute neutrophil count <1.5x10^9^/L) was observed in 4.1% (6 [n=145]) patients. Clinical profile of patients with neutropenia was not found to be different from other patients. However, *Pseudomonas aeruginosa* was cultured from three of these six patients (50%) - from blood culture in two and from pus culture in one.

Miscellaneous clinical manifestations are summarized in **Table 1**.

**Table 1:** Miscellaneous clinical manifestations in patients with X-linked agammaglobulinemia (XLA).

S. no.	Clinical manifestation	Number of patients
1.	Kawasaki disease	2
2.	Brain abscess	1
3.	Spondylodiscitis	1
4.	Psoas abscess	1
5.	Bacillus Calmette–Guérin (BCG) site ulceration and BCG adenitis*^a^*	1
6.	Pulmonary tuberculosis*^b^*	4
7.	Anterior horn myelitis	1
8.	Haemolytic Uremic syndrome (HUS)	1
9.	Immune thrombocytopenia	3
10.	Pyo-pericardium	1
11.	Vaccine associated paralytic poliomyelitis (VAPP)	1
12.	Pyoderma gangrenosum-like ulcer possibly caused by *Helicobacter sp.* *^c^* (**Figure 4**)	1
13.	Growth hormone deficiency*^d^*	1

a BCG is administered universally at birth in India. BCG adenitis is a common complication and may not be related to underlying XLA.

b There is likely no causal relationship between XLA and pulmonary tuberculosis as the latter is endemic in the region.

c Helicobacter sp. could not be grown from skin. However, patient responded to 6 months of therapy with a combination of azithromycin and doxycycline.

Skin biopsy showed dense neutrophilic rich dermal infiltrates and no evidence of an infective etiology (Gram stain, Grocott stain, PAS stain, ZN stain and modified ZN stain negative). Immunofluorescence showed negative IgG, IgM, IgA and C3.

d Received growth hormone therapy.

**Figure 4:**
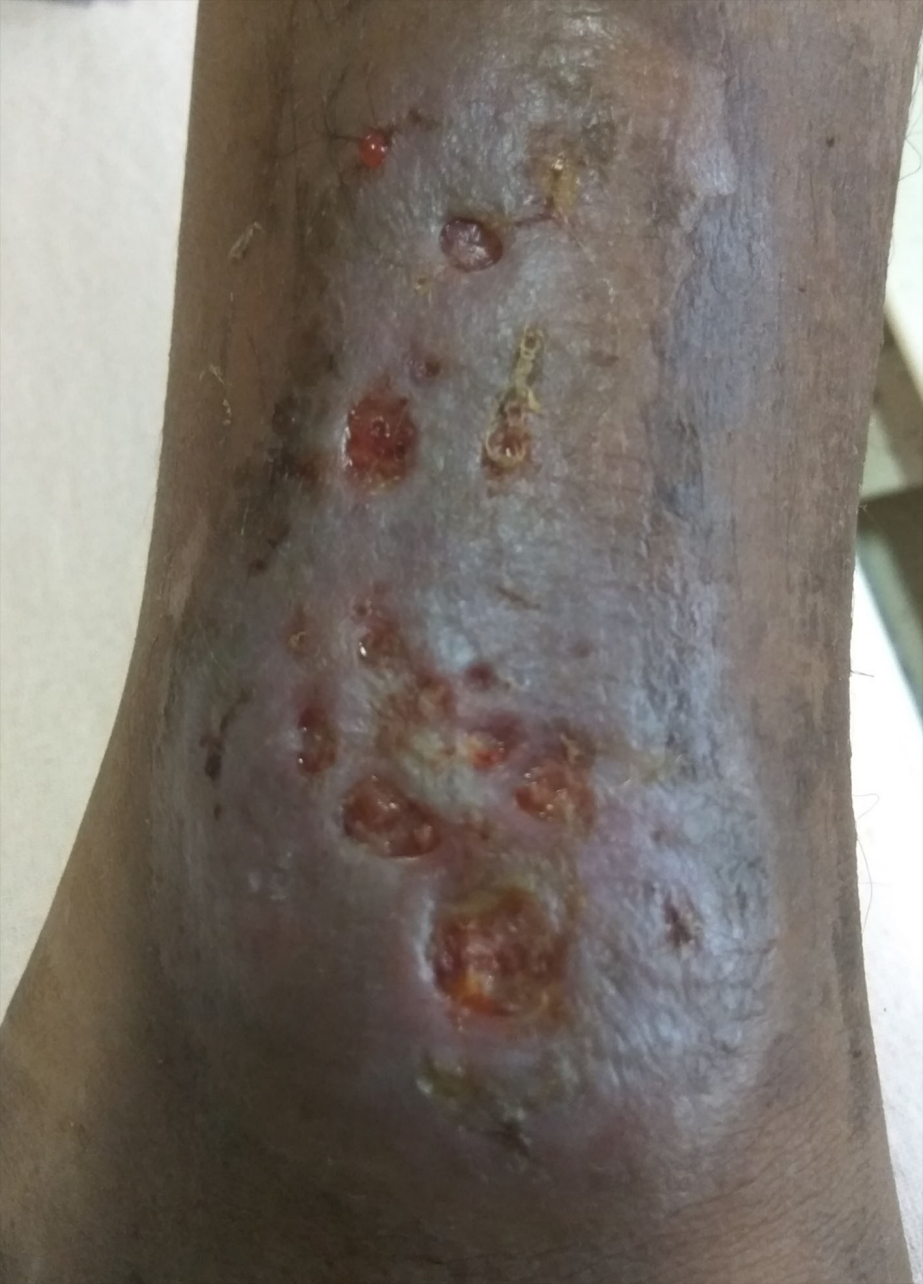
Clinical photograph of ulcerative skin lesion of a patient with X-linked agammaglobulinemia (XLA) suspected to be related to *Helicobacter sp.* infection.

### Microbiological Profile

Body fluid cultures were obtained from sites of overt infection. These included pus (from ear discharge, empyema/effusions, skin/soft tissue infections) in 29% (21/73), blood cultures in 18% (13/73) and bronchoalveolar lavage/endotracheal aspirate cultures in 8% (6/73) (**Figure 3C**). *Giardia lamblia*, *Entamoeba histolytica* and *Cyclospora spp.* were identified in stool microscopy examination in 9.5% (7/73).

An organism was identified in 37.9% [55] [n=145] patients (66 episodes of bacterial infections and seven episodes of parasitic infections).

*Pseudomonas aeruginosa* was the commonest bacterial pathogen identified (29% [19] [n=66]), followed by *Streptococcus pneumoniae* (18% [12] [n=66]), *Staphylococcus aureus* (15% [10] [n=66]) and *Klebsiella pneumoniae* (14% [9] [n=66]) (**Figure 3B**). About one-third of *S. aureus* infections were caused by MRSA (30% [3] [n=10]). Among parasitic infections, *Giardia lamblia* was identified in five patients, and *Entamoeba histolytica* and *Cyclospora spp.* were identified in one patient each.

### Immunoglobulin Profile

Levels of IgG, IgA, and IgM were reported to be below the lower limit of detection in 45.5% [66] [n=145], 68.8% [88] [n=128], and 51.5% [67] [n=130] respectively. Hence, an absolute level of immunoglobulins for these patients was not available. In the remaining patients, median IgG, IgA, and IgM levels were 1.20 (0.52, 2.90), 0.18 (0.06, 0.25), and 0.20 (0.10, 0.45) g/L respectively. Normal IgA and IgM were seen in 0.8% [1] and 8.3% [12] respectively; elevated IgA and IgM were seen in 2.3% [3] and 1.5% [2] respectively. This group with normal or elevated IgM or IgA levels comprised of 17 patients (one patient had elevation of both IgA and IgM). There was no significant difference in the mutational profile, B-cell counts, proportion of patients with undetectable IgG levels, or outcome when the group with normal or elevated IgM or IgA levels was compared to the group with low IgA and IgM. However, in patients with detectable IgG levels, the group with normal or elevated IgA or IgM had IgG levels of 4.00 (1.86, 4.94) g/L as compared to IgG levels of 1.00 (0.47, 2.75) g/L in the group with low IgA and IgM (p = 0.008, Mann-Whitney test). Serum IgE levels were measured in 53 patients and reported to be below the lower limit of detection in 53% [28]. Median IgE level in remaining patients was 5.30 (4.15, 26.25) kIU/L. Serum IgE levels were reported to be elevated in 3.7% [2].

### Lymphocyte Immunophenotyping

In our multcentric cohort, the marker panel utilized for performing basic lymphocyte immunophenotyping consisted of CD45 (Leukocyte common antigen), CD3 (T-cells), CD19 (B-cells), and CD56 (NK-cells). Btk protein expression was analyzed on CD14^+^ monocytes and was run as a separate experiment.

Median percentages of CD3^+^ T, CD19^+^ B, and CD16^+^56^+^ NK cells (of the total lymphocytes) were 92.58 (89.00, 95.75) [n=93], 0.07 (0, 0.8) [n=140], and 4.60 (3.00, 7.07) [n=67] respectively. Median absolute counts (×10^9^/L) for CD3^+^ T, CD19^+^ B, and CD16^+^56^+^ NK cells were 4.42 (2.35, 5.90) [n=66], 0.001 (0, 0.005) [n=70], and 0.21 (0.11, 0.36) [n=49] respectively. Data on Btk protein expression were available in 42 patients with median percentage positivity (percentage of monocytes expressing Btk by flowcytometry) of 17.7 (1.6, 59.0). However, the data on MFI was available in only one-third of these 42 patients.

Median percentage of CD4^+^ and CD8^+^ T cells (of the total lymphocytes) was 45.47 (37.00, 57.58) [n=27] and 41.02 (30.00, 46.46) [n=27] respectively. Median CD4:CD8 ratio was 1.22 (0.82, 1.90) [n=27]. Median absolute CD4^+^ and CD8^+^ T cell counts (×10^9^/L) were 2.02 (1.42, 3.22) [n=27] and 1.81 (1.09, 3.33) [n=27] respectively. Absolute CD4^+^ T cell counts <1.000×10^9^/L were seen in two patients aged between 1–5 years; however, none had counts <0.500×10^9^/L.

### Molecular Analysis

**Figure 5:**
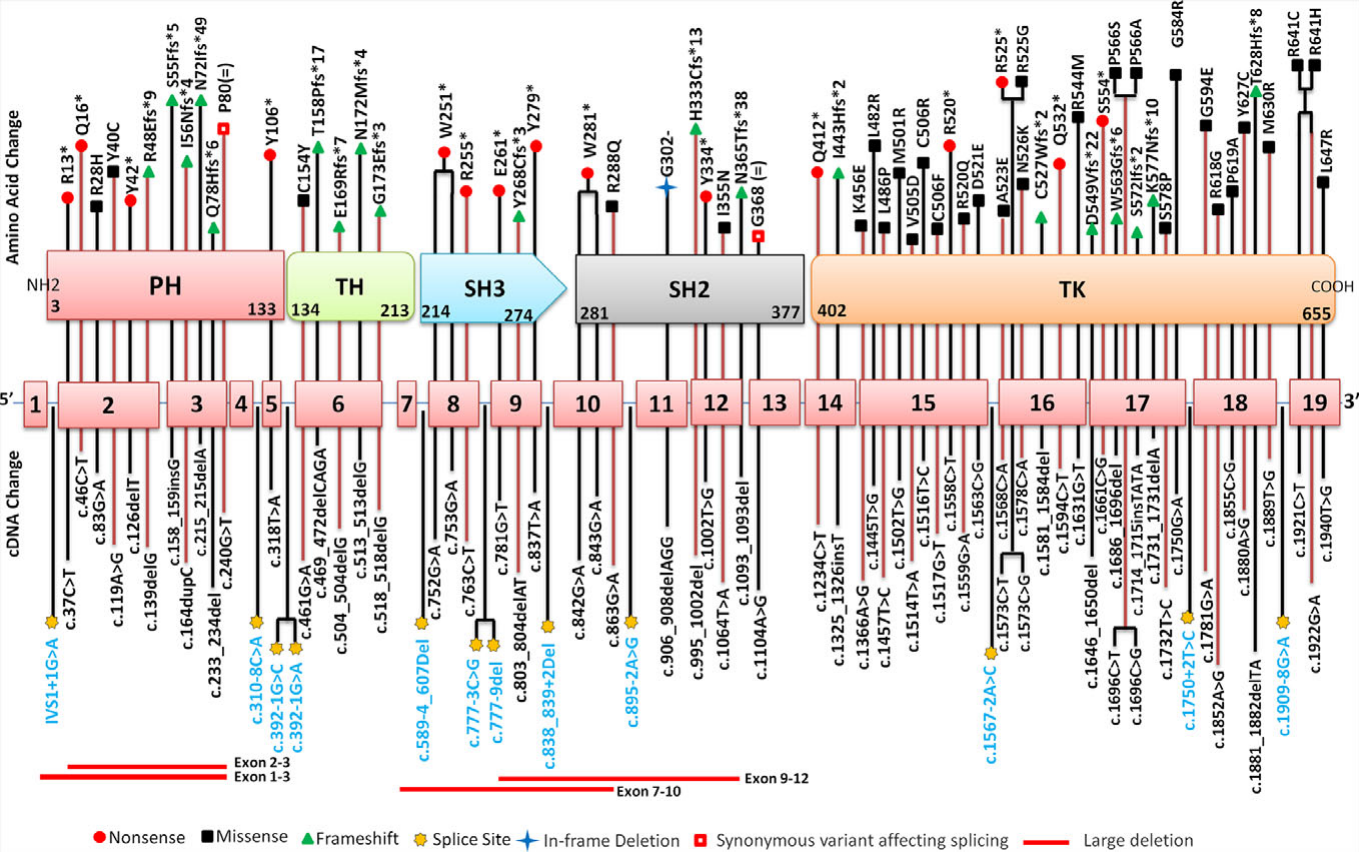
Distribution of pathogenic variants on various exons, exon-intron junctions and corresponding domains of *BTK* gene. cDNA is mentioned in the lower panel while amino acid change is mentioned in the upper panel. Corresponding amino acid changes in the Bruton tyrosine kinase (BTK) protein domains has also been highlighted using various symbols. PH, Pleckstrin homology domain; TH, Tec homology; SH, Src homology; TK, Tyrosine Kinase.

**Figure 6:**
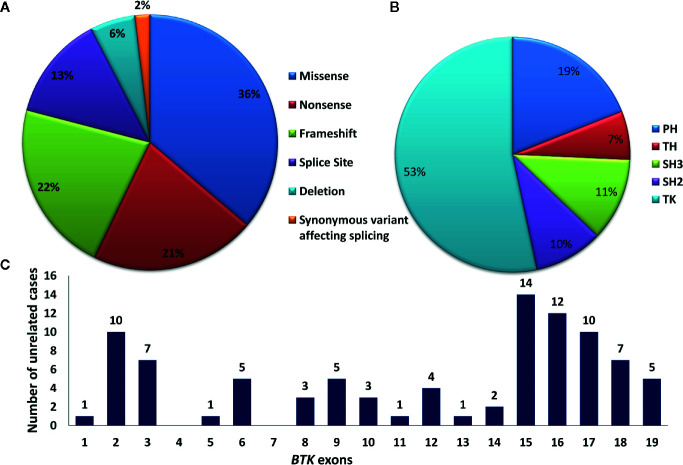
**(A)** Pie chart showing type of mutations in the *BTK* gene. **(B)** Pie chart showing affected domains on the *BTK* gene. **(C)** Bar chart showing number of variants located over individual exons of *BTK* gene. BTK, Bruton tyrosine kinase; TK, tyrosine kinase domain; PH, Pleckstrin homology; TH, Tec homology; SH, Src homology.

Molecular analysis for *BTK* gene was available for 111 patients. Of these, four were siblings or maternal cousins whereas two comprised a patient with his maternal uncle. Molecular analysis revealed 86 pathogenic variants in 105 unrelated cases. Of these, 90 patients (86%) had variants in coding part of the gene whereas 15 (14%) had intronic splice-site variants. All 86 coding and non-coding *BTK* gene variants are depicted in **Figure 5**. Missense variants were the most common [38/105 (36%)] followed by frameshift [23/105 (22%)] and nonsense variants [22/105 (21%)]. Frameshift variants due to small deletions were seen in 17.14 % (18 cases), whereas nucleotide insertions occurred in 3.80% (4 cases). Duplication induced frameshift variant was detected in 1 case (**Figure 6A**). Two synonymous exonic variants, i.e., c.240G>T; p.80G(=) and c.1104 A>G; p.368G(=) were also detected. However, these variants had a pathogenic effect by virtue of their being proximal to donor splice site and distal to the acceptor splice site respectively thereby adversely affecting splicing. Although variants were found to be distributed throughout the coding and non-coding regions of the *BTK* gene, most variants [53% (56/105)] were located in the distal exons (exon 14–19) encoding for the tyrosine kinase domain. (**Figure 6B**). Of all exons of *BTK* gene, most patients had variants in exon 15 [15/105 (14.28%)] (**Figure 6C**). In addition, 15 intronic splice- site variants were also observed - five of these variants were located in distal portion of *BTK* gene. Of the 86 variants, nine were novel (**Table 2**) and the remaining 77 had been reported previously.

**Table 2:** Novel variants seen in patients with X-linked agammaglobulinemia (XLA).

S. no.	Variant	Variant Change
1.	c.158_159insG	S55Ffs*5
2.	c.1325_1326insT	F442fs*444
3.	c.164dupC	I56Nfs*4
4.	IVS6-1C>T	c.392-1G>A
5.	c.518_518delG	G173Efs*3
6.	c.513_513delG	N172Mfs*4
7.	c.233_234delAG	Q78Hfs*6
8.	c.1646_1650delATGAA	D549Vfs*22
9.	c.1104 A>G	G368(=)

Founder variants could not be suspected based on the data. Two variants (c.1581_1584delTTTG and c.1922G>A) were found four cases each. Five variants (c.1559G>A; c.1594C>T; c.1696C>T; c.215_215delA and IVS18-8G>A) were detected in three cases each. In the group with normal or elevated IgA or IgM, mutational details were available in 59% [10] [n=17] and are as follows: c.392-1G>C; c.1516T>C, p.C506R; c.1567-2A>C; c.1581_1584delTTTG, p.C527Wfs*2; c.1594C>T, p.Q532*; c.215_215delA, p.N72Ifs*49; c.752G>A, p.W251*, c.83G>A, p.R28H; Del-exon 9-12; IVS1+1G>A.

### Genotype-Phenotype Analysis

Patients with missense mutations (36% [40] [n=111]) had 11 episodes of infection in which an organism could be identified, whereas, patients with other non-missense mutations (non-sense, frameshift, splice-site, deletions) had 43 such episodes. This translated to a 2.8-fold (95% CI 1.2–6.8, p=0.02) higher risk of identification of an organism in patients with non-missense mutations as compared to patients with missense mutations. Additionally, *Pseudomonas aeruginosa* infection was seen almost exclusively in patients with non-missense mutations. However, there was no significant difference in the syndromic diagnosis (pneumonia, diarrhea, otitis media, etc.) of infections. There was no difference in the age at onset of symptoms, age at diagnosis, the duration of delay in the diagnosis, B-cell proportions, or immunoglobulin levels between the two groups.

When mutations were stratified as per their severity (based upon previous reports or *in-silico* predictions), patients with less severe mutations (7.2% [8] [n=111] had 2 episodes of infection in which an organism could be identified, whereas patients with severe mutations (92.8% [103] [n=111]) had 52 such episodes. However, there was no difference in the age at onset of symptoms, age at diagnosis, duration of delay in the diagnosis, B-cell proportions, or immunoglobulin levels, or overall survival between the two groups. Extensive genotype-phenotype correlation could not be carried out in our cohort due to lack of data regarding Btk protein expression (data regarding Btk protein expression was available in only 28.9% of the cohort).

### Treatment and Outcome

In India, all patients with XLA are presently being treated with intravenous immunoglobulin (IVIg, at a dose of ~400 mg/kg/month) as subcutaneous immunoglobulin is not available. Many patients had difficulties in accessing this therapy because of financial reasons and lack of universal health insurance in India. Therefore, IVIg could not be given to several patients at recommended doses and intervals. Breakthrough infections were managed using antimicrobials based on culture reports and/or clinical judgment of treating physicians. Cotrimoxazole prophylaxis (5 mg/kg/day of trimethoprim component) is also prescribed to several patients. Many patients with XLA in India who are on replacement IVIg, are being supported by FPID, various state governments and philanthropic organizations. In addition, a few patients were procuring IVIg on their own. Four patients in the cohort also underwent hematopoietic stem transplantation (HSCT) at Apollo Hospitals, Chennai. None of these four patients were on regular IgRT prior to HSCT. The decision to transplant was based on the caregivers’ preferences (life-long IgRT was considered to be more cumbersome and costly as compared to one time potentially curative treatment, although, with significant risk of mortality). In our setup, the cost of life-long IgRT is approximately 7–10 times the cost of HSCT. The details of the transplant procedure have been presented in **Supplementary Table 1**.

**Figure 7:**
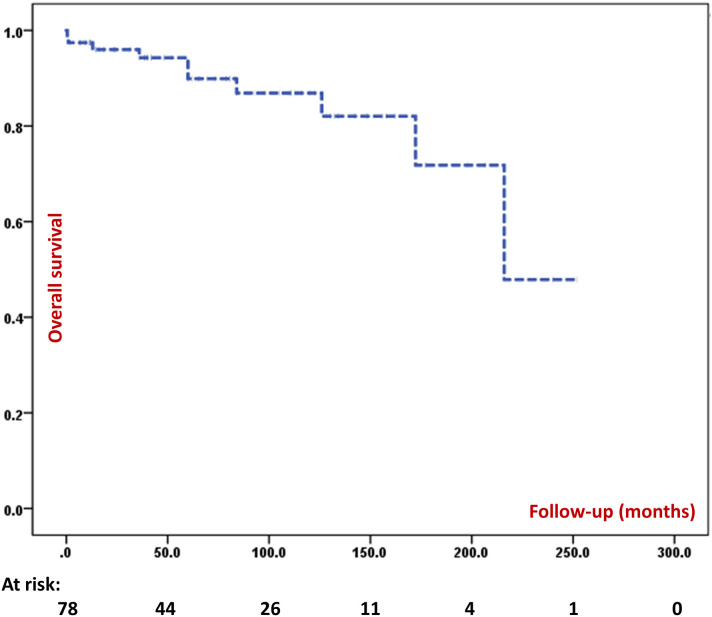
Kaplan Meir survival curve of the entire cohort.

Follow-up details were available for 108 patients. Of these, 12% [13] had died till the time of this analysis (four patients died in the 1^st^ decade, seven patients during 2^nd^ decade and two patients during 3^rd^ decade). We calculated 5-year and 10-year survival in patients for whom the follow-up data were available. The 5-year survival was 89.9% and 10-year survival was 86.9%. **Figure 7** depicts the survival curve of 108 patients in the present cohort. Thirty-four patients are surviving beyond the age of 10 and nine patients are surviving beyond the age of 20. The 20-year survival was 47.9%, however, only two patients in the entire cohort have been followed-up for 20 years or more. Median duration of follow-up was 61.0 (24.0, 120.6) [n=78] months and total duration of follow-up was 6083.2 patient-months (506.9 patient-years) [n=78]. All four patients who underwent HSCT had attained B cell reconstitution and were doing well at 7, 15, 18, and 48 months of follow-up. None of these four patients were receiving IVIg replacement at time of this analysis.

## Discussion

We report a multicenter cohort of 145 patients with XLA from India. As there is no national registry for IEIs in India, collection of data on various PIDs from different centers may give information on nationwide burden of these diseases.

Median age at onset of symptoms and diagnosis was 1 and 5 years respectively with a median delay in diagnosis 3.5 years. In our previous study we had reported a mean delay of 4.2 years Delays in diagnosis are primarily a reflection of lack of awareness about PIDs among medical professionals as well as the laity. Diagnostic delays of upto several years have been noted in series from Latin America, Africa and the Middle East (24–27) (**Table 3**). However, a greater delay in the diagnosis was noted in our scenario (20) emphasizing the need for concerted efforts to shorten this delay.

**Table 3:** Comparison of clinical profile of patients with X-linked agammaglobulinemia (XLA) in the present series from previously reported studies.

S. no.	Author (Country), Year, (Reference)	No. of patients	Age at presentation (years)	Age at diagnosis (years)	Family history	Pneumonia	Diarrhea	Skin and soft tissue infections	Arthritis	Neutropenia	Meningitis	Encephalitis	Most common organism	Mortality
1	Rawat et al. (India), Present study	145	1 (median)	5 (median)	60%	82.6%	42.4%	35.4%	26%	4.1%	23%	4.1%	*Pseudomonas aeruginosa* (overall) Giardia (diarrhea)	13%
2	Winkelstein et al. (USA), 2006 (28)	201	<1 (50% patients)	<2 (50% patients)	41%	62%	23%	18%	7%	11%	12%		Pneumococcus (pneumonia) Giardia (diarrhea) Pseudomonas (sepsis)	8.5%
3	Chen et al. (China), 2016 (29)	174	1 (median)	7.09 (mean)	34.71%	77%	18.97%	8.05%	18.75%	5.17%	19.65%	NR	NR	
4	Lougaris et al. (Italy), 2020 (22)	168	NR	7 (mean) before 2000; 23 months (mean) after 2000	39.3%	39.9%	19%	20.8%	9.5%	NR	4.8%	0.6%	*Hemophilus influenzae* followed by *Streptococcus pneumoniae*	7.7%
5	Plebani et al. (Italy), 2002 (30)	73	1 (median)	3 (median)	39.7%	53.4%; 50% (after IVIg))	13% (4% after IVIg)	27%	10%	1%	4%	1% (after IVIg therapy)	NR	1.4%
6	Lee et al. (Hong Kong), 2010 (31)	62	1 (median)	7 (median)	35%	72.6%	29%	25.8%	29%	NR	12.9%		NR	NR
7	Basile et al. (Argentina), 2009 (24)	52	<1 (>50% patients)	3.5 (median)	55%	61%	27%	31%	8% (osteo-articular)	22%	16%		Pneumococcus (pneumonia) Giardia (diarrhea) Pseudomonas (sepsis)	6.1%
8	Aadam et al. (2016), North Africa (25)	50	9 months (median)	3 (median)	32%	55%	42%	15%	37.5%	NR	27.5%		*Pseudomonas aeruginosa* (overall)	NR
9	Aghamohammadi et al. (Iran), 2006 (26)	37	10 months (median)	4 (median)	52%	86%	78%	NR	24%	2.5%	24%	11%	NR	14%
10	Singh et al. (India), 2016 (9)	36	1 (median)	5 (median)	57%	86%	44%	25%	42%	11%	25%		NR	19%
11	Moin et al. (2004), Iran (32)	33	8 months (median)	4 (median)	NR	81.8%	75.8%	6.1%	21%	3%	24.2%	12.1%	NR	15%
12	Esenboga et al. (Turkey), 2019 (33)	32	9.5 months (median)	3.5 (median)	47%	53.1%	53.1%	3.1%	6.2%	9.3%	8.3%	12.4%	NR	NR
13	Yeh et al. (2020), Taiwan (34)	29	1.2 (median)	5 (median)	NR	68.4%	13.8%	17.2%	3.4%	3.4%	3.4%	NR	*Pseudomonas aeruginosa* (overall)	6.8%
14	García et al. (2016), Mexico (35)	26	NR	NR	NR	69.2%	19.2%	23%	38.4%	NR	38.5%	NR	*Pseudomonas sp.* (overall)	NR
15	Dogruel et al. (2019), Turkey (27)	22	5 months (median)	15.5 months (median)	68.1%	86.3%	36.4%	NR	18%	NR	NR	4.5%	NR	0%

IVIg, intravenous immunoglobulin; NR, not reported.

In the present study, pneumonia and other respiratory tract infections and otitis media were the most common clinical manifestations. Similar results have also been reported in several other cohorts including one previously reported series from Chandigarh, North India (9, 22, 24, 26, 28, 30, 31) (**Table 3**).

Arthritis was seen in 26% patients in the present cohort. Arthritis is an important clinical manifestation in patients with XLA. It may be infectious or non-infectious and in the latter case may mimic juvenile idiopathic arthritis (JIA) (15, 36). Six patients in present cohort had polyarthritis—one among these has been reported previously (15). In a previously reported single center series on XLA from Chandigarh, arthritis was observed in 42% patients. Joint involvement in patients with XLA in present series was observed to be higher than that reported from United States, Argentina, Italy and Iran (24, 26, 28, 30) but similar to that reported from Hong Kong (29%) (31, 37), Mexico (38%) (35), Africa (37%), Turkey (22%) (27) and China (22%) (29) (**Table 3**). It has been observed that patients with XLA who fail to maintain adequate trough levels despite IVIg replacement therapy are at higher risk of developing arthritis. A study published from Chandigarh, India in 2017 measured the serial trough levels in patients with XLA. In this study, it was observed that the mean trough IgG level (435 mg/dl) is much less as compared to the trough levels of IgG that are seen in western countries (38). This could have been a reason for higher proportion of children with arthritis seen in our cohort. Another likely reason for higher proportion of children with arthritis is delay in diagnosis of XLA in India.

Patients with XLA are predisposed to develop pyogenic infections caused by *Streptococcus pneumoniae, H. influenzae, S. aureus* and *P. aeruginosa.* In the present cohort, *Pseudomonas aeruginosa* was the most common organism isolated followed by *Streptococcus pneumoniae*. While *Pseudomonas aeruginosa* is not a signature organism for patients with XLA, it has been found that patients with XLA who have episodes of neutropenia are at increased risk of pseudomonas sepsis (20). In a recently reported series from Taiwan, Pseudomonas sepsis was the most common clinical presentation (34). Winkelstein et al. reported data on XLA from United States registry in 2006. In this study, commonest causes of sepsis were *Pseudomonas sp.* (29%) and *Streptococcus pneumoniae* (24%) (28). However, the most common cause of bacterial infection was *Streptococcus pneumoniae* followed by *Haemophilus influenzae*. Similar results were also reported by Basile et al from Argentina (24). In a previously reported series from India (9), only one of 36 patients had *Pseudomonas aeruginosa* as the etiological agent for sepsis. Similarly, in a study from Mexico by García et al. (35), *Pseudomonas sp.* was identified in 1/12 patients as a cause of ecthyma gangrenosum. In our multicenter cohort, we observed an unusually high percentage of patients with XLA in whom *P. aeruginosa* was identified as a cause of any infection. Even though neutropenia has been identified as a risk factor for pseudomonas sepsis in patients with XLA, only 4% patients in the present series had neutropenia. Nonetheless, pediatricians, internists, and hematologists evaluating children for neutropenia should consider XLA among the differential diagnosis.

**Figure 8:**
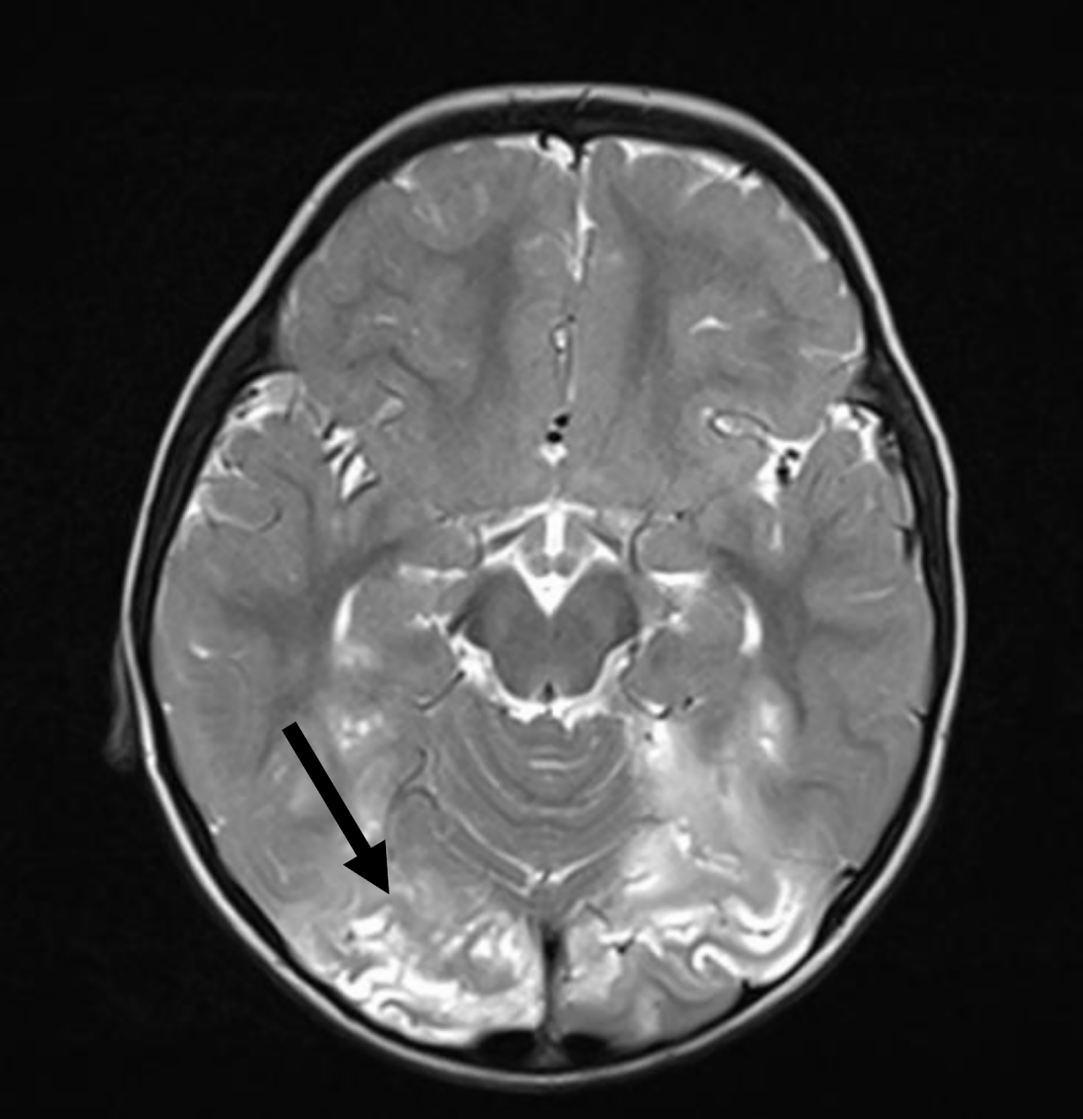
Magnetic resonance imaging shows T2-weighted cortical and subcortical hyperintensities in bilateral occipital region.

Although, viral infections are not usually seen in patients with XLA, viral encephalitis (often caused by polio and non-polio enteroviruses) is an uncommon but potentially life-threatening complication. In an international survey of patients with XLA reported in 2019, this complication was seen in 4.6% of patients. Meningoencephalitis has been observed in up to 1/3^rd^ of all patients with XLA in various series. However, etiological agent is not identified in most cases (9, 20, 24, 26, 28, 30, 31). In the present cohort, 4.8% patients developed encephalitis. The etiology for encephalitis could not be determined in most and diagnosis was largely based on clinical and radiological findings (**Figure 8**). However, in one patient who had fatal encephalitis, the brain specimen on autopsy showed features suggestive of enteroviral encephalitis (39). In another patient, *S. pneumoniae* was the cause of meningoencephalitis like presentation. VAPP was seen in one patient from Mumbai, West India. VAPP is still a significant problem for patients with primary B cell and combined immunodeficiency in countries such as India, where live oral polio vaccine is being used (21).

Other viral infections have rarely been reported in patients with XLA (40–46). Extensive molluscum contagiosum infection was seen in one patient in the present series (12).

Mycobacterial infections are not usually seen in patients with XLA. It has been observed in patients with XLA from Argentina and India (20). In the present cohort, four patients developed pulmonary tuberculosis and one had BCG adenitis (**Table 1**).

Autoimmune and inflammatory complications have also been reported in patients with XLA (2). Common ones include inflammatory arthritis, inflammatory bowel disease, thrombocytopenia and Kawasaki disease (11). In the present series, inflammatory arthritis was seen in six patients, immune thrombocytopenia in four, haemolytic uremic syndrome in one and Kawasaki disease in two (11).

In our cohort, missense variants in *BTK* gene involving tyrosine kinase domain were the most common mutations. Similar results have also been reported from China, Africa, Hong Kong, Argentina and Italy (22, 24, 25, 29, 31) (**Table 4**). Most variants were located in exon 15 as has also been reported from China and Italy (22, 29). However, most variants were found to be located on exon 2 in a study from Hong Kong (29). Nine of 86 variants in our study were novel.

**Table 4:** Comparison of genetic profile of patients with X-linked agammaglobulinemia (XLA) in the present series from previously reported studies.

S.no	Author (Country), Year, (Reference)	No. of variants ((*) = genotype-phenotype correlation available)	Types of mutations	Most commonly affected domain of *BTK* gene	Most commonly affected exon of *BTK* gene	Novel variants
1	Rawat et al. (India), present study	86 variants from 105 patients (*)	Missense mutations (37%) Frameshift mutations (21%) Splice-site mutations (14%) Nonsense mutations (21%) Deletions (5%) Synonymous variants affecting splicing (2%)	TK (55%) PH (19%) TH (6%) SH3 (11%) SH2 (9%)	15	9
2	Chen et al. (China), 2016 (29)	127 variants from 124 families were found to have *BTK* gene mutation (*)	Missense mutations (n=48) Frameshift mutations (n=29) Splice-site mutations (n=23) Nonsense mutations (n=21) Deletions (n=5)	TK domain (n=64) PH domain (n=28) SH2 domain (n=16) SH3 domain (n=15) TH domain (n=3)	15	45
3	Lougaris et al. (Italy), 2020 (22)	104 different variants from 125 families (*)	Missense mutations (49%) Indels (18%) Nonsense mutations (17%) Splice-site mutations (12%) Large deletions (4%)	NR	15	18
4	Broides et al. 2006, USA (47)	94 different variants (*)	Missense mutations (36.4%) Frameshift mutations (10%) Premature stop codons (15.5%) Splice-site mutations (25.4%) Gross deletions (5.4%) Duplication/inversions (2.7%) Complex mutations (3.6%) Retroposon insertion (0.9%)	NR	NR	NR
5	Lee et al. (Hong Kong), 2010 (31)	56 different variants in 57 families (*)	Missense mutations (n=20) Insertion mutations (n=1) Splice-site mutations (n=12) Nonsense mutations (n=6) Deletions (n=13) Promoter (n=1) Complex mutations (n=3)	TK domain (n=23) PH domain (n=16) SH2 domain (n=12) SH3 domain (n=4) TH domain (n=1)	2	15
6	Aadam et al. (2016), North Africa (25)	33 different variants from 35 families (*)	Missense mutations (n=12) Frameshift mutation (n=4) Splice-site mutations (n=6) Nonsense mutations (n=6) Deletions (n=2), in frame deletion (n=1), complex mutation (n=2; one indel and one double mutation)	TK domain (n=17) PH domain (n=6) SH2 domain (n=4) SH3 domain (n=3) TH domain (n=2) 1 frameshift mutation was predicted to affect both SH2 and TK.	NR	17
7	Basile et al. (Argentina), 2009 (24)	29 different variants from 35 families (*), *^a^*	Missense mutations were most common followed by non-sense mutations	NR	NR	9
8	Aghamohammadi et al. (Iran), 2006 (26)	18 different variants in 21 families (*), *^a^*	Missense mutations (n=7) Splice-site mutations (n=5) Nonsense mutations (n=3) Deletions (n=3)	TK domain (n=10) PH domain (n=4) SH2 domain (n=1) SH3 domain (n=1) TH domain (n=1) SH1 domain (n=1)	NR	13
9	García et al. (2016), Mexico (35)	12 different variants (*),*^b^*	Missense mutations (n=4) Splice-site mutations (n=7) Deletion mutation (n=1)	SH2 (n=6) SH1 (n=3) PH (n=1) SH3 (n=1) One mutation located in both SH1 and SH2	NR	4
10	Dogruel et al. (2019), Turkey (27)	12 variants from 12 families (*), *^a^*	Missense mutations (n=3) Splice-site mutations (n=3) Nonsense mutations (n=4) Deletions (n=2)	PH domain (n=2) SH1 domain (n=6) SH2 domain (n=2) SH3 domain (n=2)	11	2

BTK, Bruton tyrosine kinase; TK, tyrosine kinase domain; PH, Pleckstrin homology; TH, Tec homology; SH, Src homology; NR, not reported.

a Genotype-phenotype analysis based on individual mutations rather than the severe-mild classification commonly used for such analysis.

b Genotype-phenotype analysis based on the domain of Btk affected.

Because of non-availability of subcutaneous immunoglobulin therapy in India, IVIg is the cornerstone of management. However, access to IVIg therapy is not easy for most patients due to high cost of treatment and absence of universal health insurance. In last 5 years, several state governments in India (e.g. Governments of Punjab, Haryana, Madhya Pradesh, Delhi, Himachal Pradesh, Karnataka, Kerala, Tamil Nadu and West Bengal) have started supporting IVIg therapy to patients with PIDs. FPID and other philanthropic organizations have been supporting the treatment of several patients with hypogammaglobulinemia across the country for last 10 years. Despite the generous support provided by aforementioned government and non-government organizations, several patients still find it difficult to get their monthly doses of IVIg. As a result of these constraints, most patients do not get the recommended doses of IVIg and fail to maintain trough levels of IgG above the recommended levels (38).

Survival rates of XLA vary from country to country. These are largely determined by availability of diagnostic facilities and ease of access to treatment. While more than 70% survival beyond the age of 20 has been reported from United States, Europe and Australia, corresponding figures are less than 40% from Asia and Africa (20). Mortality rate in the present cohort was 12%. Prognosis of XLA in the developed countries is much better (e.g. 8.5% mortality United States in 2005 (28); 6.1% in Argentina in 2009 (24) and 1.4% in Italy in 2002) (30) (**Table 3**).

There are several difficulties in diagnosis and management of XLA in India. Because of lack of awareness, several patients are diagnosed late and have already developed chronic lung disease by the time they start receiving their immunoglobulin replacement therapy. Diagnostic facilities such as nephelometry, flow cytometry and genetic sequencing are available only in tertiary level institutes or medical schools. Access to IVIg is not easy for several of these families and most patients do not get recommended doses. As a result of this, patients present with frequent breakthrough infections that adds to their morbidity and mortality. Despite all difficulties, it is noteworthy that 9 of the patients in the present series have now reached their 3^rd^ decade of life and are doing well on immunoglobulin replacement therapy.

HSCT was performed in four patients at a single center with expertise in transplant facility (48, 49). There have been rare reports of successful HSCT in patients with XLA (50, 51). All 4 children in the present series underwent successful HSCT and are off IVIg. HSCT has been performed sparingly in patients with XLA. The conditioning regimens and the outcomes have been variable (52). As noted in our scenario, HSCT utilizing treosulfan-based conditioning regimens have provided promising outcomes. Till date, reports describing successful gene therapy in patients with XLA are lacking. However, studies on gene therapy in murine models of XLA have shown encouraging results with full B-cell recovery (53).

Strengths of this study are that this is the first multicenter cohort on XLA from India and one of the largest cohorts in world. Limitations of this study are that complete data were not uniformly available from all centers. Details of molecular analysis were not available for approximately 20% patients. In India, as there is a disparity in the availability of diagnostic testing, recognition of important clinical findings (absence of tonsils and lymph nodes) is essential for diagnosing XLA. However, data on these findings was available from only a few centers due to the retrospective design of our study.

## Conclusion

We report a multicenter cohort of patients with XLA from India. *Pseudomonas aeruginosa* was the most common organism isolated, even in the absence of neutropenia. VAPP is still a significant problem for patients with primary B cell and combined immunodeficiency in countries where live oral polio vaccine is being used. In addition, few patients in this XLA cohort also had mycobacterial infections. However, India being endemic for tuberculosis, it would be difficult to ascribe these mycobacterial infections to the underlying antibody defect. There is a significant delay in the diagnosis of XLA because of a lack of awareness among pediatricians and internists. Delay in the diagnosis does not only lead to a greater burden of infection, but it may also result in organ damage and a higher burden of autoimmune complications in patients with XLA. Facilities for molecular confirmation of diagnosis are not available at many centers in the country. Provision of replacement immunoglobulin therapy is a daunting task for families with affected children. However, with financial support provided by FPID; some state governments in India and several philanthropist organizations, several patients have been able to access immunoglobulin replacement therapy now.

## Data Availability Statement

The raw data supporting the conclusions of this article will be made available by the authors, without undue reservation.

## Ethics Statement

Ethical review and approval was not required for the study on human participants in accordance with the local legislation and institutional requirements. Written informed consent to participate in this study was provided by the participants’ legal guardian/next of kin.

## Author Contributions

AR, AKJ: Data collection, writing of initial draft, editing of manuscript at all stages of its production, patient management, review of literature. DS, PV, AG, BS, RM, MS, MD, PT, AP, VG, SS-D, MG, AD, MM, AA, RR, RU, SB, AJ, HL, LR, DM, MK, AS: Data collection, management of patients, review of the final manuscript. AB, RT, KA, VJ, SM, JS: Data collection, writing of initial draft, review of literature. RSa, RSh, RG: Patient management, review of literature. KI, SN, OO, PL, KC, Y-LL: Genetic evaluation, review of the final manuscript. SS: Data collection, patient management, review of literature, editing and critical revision of manuscript at all stages of its production, final approval of manuscript. All authors contributed to the article and approved the submitted version.

## Funding

Indian Council of Medical Research (ICMR), New Delhi, India, and Department of Health Research, Ministry of Health and Family Welfare, Government of India, New Delhi, India for funding (vide Grant No. GIA/48/2014-DHR). However, the funders had no role in study design, data collection and analysis, decision to publish, or preparation of the manuscript.

## Conflict of Interest

The authors declare that the research was conducted in the absence of any commercial or financial relationships that could be construed as a potential conflict of interest.
