# Supplementary material for: Clinical and Genetic Profile of X-Linked Agammaglobulinemia: A Multicenter Experience From India
Source: Front Immunol. 2021 Jan 15;11:612323. doi: 10.3389/fimmu.2020.612323 (PMC7873890; doi:10.3389/fimmu.2020.612323)
Supplement: Supplementary file 1 [file Table_1.docx]

**Supplementary Table 1:** Details of donor selection, conditioning regimens, transplant-related complications, and outcome of 4 patients who underwent hematopoietic stem cell transplantation in out cohortat Apollo Hospitals, Chennai

| **Donor** | **Conditioning** | **Complications** | **Chimerism status** | **Follow-up** |
| --- | --- | --- | --- | --- |
| Matched sibling | Thiotepa, treosulphan, fludarabine | Skin GVHD - resolved | Complete | Well |
| Matched sibling | First transplant: fludarabine, busulphan  Second transplant - thiotepa, treosulphan, fludarabine | Rejection of first transplant | Complete after second transplant | Well |
| Haploidentical stem cell transplantation from father | Fludarabine, treosulphan | CMV viremia, retinitis, | Mixed | Neurocognitive and visual sequelae |
| Matched sibling | Thiotepa, treosulphan, fludarabine | Nil | Complete | Well |
